# Dietary Administration of the *Bacillus subtilis* Enhances Immune Responses and Disease Resistance in Chickens

**DOI:** 10.3389/fmicb.2020.01768

**Published:** 2020-07-22

**Authors:** Mengjiao Guo, Mingtao Li, Chengcheng Zhang, Xiaorong Zhang, Yantao Wu

**Affiliations:** ^1^Jiangsu Co-innovation Center for Prevention and Control of Important Animal Infectious Diseases and Zoonoses, College of Veterinary Medicine, Yangzhou University, Yangzhou, China; ^2^Joint International Research Laboratory of Agriculture & Agri-Product Safety (JIRLAAPS), Yangzhou University, Yangzhou, China

**Keywords:** *Bacillus subtilis*, growth performance, intestinal microbiota, immunity response, *Escherichia coli*

## Abstract

*Bacillus subtilis* (*B. subtilis*) has a variety of proposed beneficial effects for chickens, including growth promotion and disease prevention. In this study, chickens were fed a diet containing *B. subtilis* for 21 days and growth performance, intestinal morphology, intestinal microbiota, immune responses, and disease resistance were investigated. After 21 days of feeding, chickens fed a diet containing *B. subtilis* had higher body weights. The concentrations of serum immunoglobulins IgA and IgM were significantly increased by *B. subtilis* in the diet. Moreover, chickens fed with *B. subtilis* had greater villus height (VH), shallower crypt depth (CD), and a higher VH/CD ratio in the jejunum than chickens fed a standard control diet. Diet with *B. subtilis* can balance intestinal microbiota, facilitate an increase in beneficial bacteria, and inhibit the pathogenic bacteria after 21 days of feeding. After an *Escherichia coli* (*E. coli*) challenge, the survival rate of chickens fed with *B. subtilis* was 66.67%, which was significantly higher than the controls. The *E. coli* contents in spleens and lungs from chickens fed a diet with *B. subtilis* were lower than those in controls. In addition, *B. subtilis* can trigger the toll-like receptor 4 and cause induction of proinflammatory cytokine (*Il1*β, *Il6*, and *Il8*) production to develop innate immune responses in chickens. In conclusion, diets containing *B. subtilis* can improve growth performance, serum immunoglobulin levels, the intestinal villus-crypt system, intestinal homeostasis, immune responses, and disease resistance against *E. coli* in chickens.

## Introduction

Since 2006, growth-promoting antibiotics have been banned in Europe. Since 2020, all forms of growth-promoting antibiotics except traditional Chinese medicines have been forbidden to be used as feed additives in China. At present, with the increase in the number of laws and regulations concerning the use of antibiotics, and the difficulty of new drugs development, thus increasing the demand for growth promoter alternatives (Lupindu et al., 2015). Probiotics are live microorganisms that confer health benefits to the host to improve nutrient digestibility, improve gut health, enhance intestinal barrier integrity, and modulate immune responses (Fuller, 1989; Lee et al., 2010; Pagnini et al., 2010; Knap et al., 2011; Abdelqader et al., 2013). It has become a safe alternative that can partly replace antibiotics. Due to the strong resistance of spore to extreme conditions and long term storage, *Bacillus* spp., as probiotics, are recognized as the most suitable probiotics (Hong et al., 2005). Recent studies have confirmed that dietary inclusion of *Bacillus* spp. have beneficial effects in chickens (Al-Fataftah and Abdelqader, 2014; Lee et al., 2015; Whelan et al., 2019).

Among the wide varieties of bacterial species used as probiotics, *Bacillus subtilis* (*B. subtilis*) has been considered to be safe and effective for animals (EFSA, 2007). *B. subtilis* is well known for its capability to improve growth performance, compete with pathogens, balance intestinal microbiota, and improve disease resistance (Elshaghabee et al., 2017; Abudabos et al., 2019). Balanced intestinal microbiota benefit the host by competing for nutritional sources and adhesion sites of pathogens, promoting commensal proliferation, and improving the intestinal immune system (Ng et al., 2009; Ma et al., 2018). In poultry, avian colibacillosis causes different syndromes in 3–12 week old broiler chickens that usually present a systemic infection with characteristic fibrinous lesions and septicemia (Schouler et al., 2012). At the outbreak period of avian colibacillosis, serious threats to poultry industry occurred. It was reported that dietary *B. subtilis*-based direct-fed microbials promoted growth performance and balanced the gastrointestinal microbiota by inhibiting the colonization of avian pathogenic *Escherichia coli* (*E.coli*) and *Clostridium perfringens* (Gebert, 2007; Lee S.H. et al., 2014). In addition, dietary direct-fed microbials alleviate clinical symptoms of ubiquitous poultry diseases, such as necrotic enteritis and Salmonellosis (Abudabos et al., 2019; Sokale et al., 2019).

Different presumable mechanisms for probiotic actions have been proposed and investigated, including inhibition and stimulation of host immunity. Innate immunity is the first-line of defense against the pathogens, and it has been demonstrated that dietary *B. subtilis* increases innate and acquired immune responses (Pagnini et al., 2010; Gadde et al., 2017). As expected, *B. subtilis* was shown to modulate host protective immune responses against bacterial infections (Rajput et al., 2014). However, the beneficial effects of *B. subtilis* are markedly strain-dependent as many properties vary as a function of strain (Larsen et al., 2014; Dowarah et al., 2018). In our previous study, *B. subtilis* BG5 and BYS2 strains were selected based on their beneficial effects in the gastrointestinal tract and inhibition of the growth of pathogenic bacteria (Guo et al., 2017). To gain better insight into the role of *B. subtilis*, the effects of dietary *B. subtilis* on growth performance, the intestinal villus-crypt system, intestinal microbiota, immune responses, and resistance to *E. coli* were investigated in chickens.

## Materials and Methods

### *B. subtilis* and Pathogen

The probiotics, *B. subtilis* BG5 and BYS2, were isolated and characterized according to a previous study (Guo et al., 2017). A single colony was cultured in nutrient broth medium (Hopebio, Qingdao, China) at 37°C for 12 h. The probiotic microbe count was 10^8^ cfu/mL.

The bacterial pathogen, *E. coli* (O1K1), was isolated from clinically infected ducks and grown in Luria-Bertani medium at 37°C for 10 h. The bacterial suspension was prepared for the infection experiment in chickens at a concentration of 10^7^ cfu/mL.

### Experimental Design

One-day-old chickens (Ross 308) were purchased from a commercial hatchery and randomly allotted to two groups. Chickens of control group were fed with standard control diet (Table 1). Chickens of *B. subtilis* group were fed with 10^6^ cfu/g *B. subtilis* (mixture of BYS2 and BG5). Each group consisted of three replicates with 30 chickens per replicate. The chickens were weighed individually, and blood samples were collected through the wing vein at days 7, 14, and 21. On days 14 and 21, five chickens were randomly selected from each replicate and euthanized. Spleens were collected and stored at −70°C for RNA extraction. On day 21, the chickens were challenged with *E. coli*. All experiments were carried out in accordance with the principles of the Basal Declaration and Recommendations of Committee on the Ethics of Animal Experiments of Yangzhou University. The protocol was approved by the Committee on the Ethics of Animal Experiments of Yangzhou University.

**TABLE 1:** Composition of the standard diet.

Ingredients	Percentage (%)	Nutrientlevels	Content (%)
Corn	66.50	ME/(MJ/kg)	11.97
Soybean meal	27.00	CP	19.06
Fish meal	2.00	Met	0.47
CaHPO_4_	1.70	Lys	0.89
Limestone	1.10	Ca	0.98
DL-methionien	0.20	TO	0.71
L-Lysine	0.20		
NaCl	0.30		
Premix^a^	1.00		
Total	100		

*^a^Premix provided the following each kilogram of diets:VA 10000 U; VB_1_ 5 mg; VB_2_ 10.0 mg; VB_6_ 8.2 mg; VB_12_ 40.0 mg; VD_3_ 6000 U; VE 50 U; VK_3_ 3.0 mg, pantothenic acid 45.0 mg; biotin 2.0 mg; folic acid 1.0 mg; Cu 8.0 mg; Fe 80.0 mg; Mn 80.0 mg; Zn 90.0 mg; I 1.5 mg; Se 0.3 mg.*

### Histomorphological Analysis

On day 21, intestinal samples from the middle part of duodenum, jejunum, and ileum were collected and submerged in 4% paraformaldehyde solution. Intestinal samples were sliced using a microtome and then stained with hematoxylin and eosin (H&E). Intestinal morphology was observed with a Leica microscope. Villus height was measured from the tip of the villus to the villus-crypt junction. Crypt depth was measured the depth of the invagination between adjacent villi. A total of 10 intact and well-oriented crypt-villus units were selected from each intestinal cross-section. The morphological measurements were made in 10-μm increments with Image-Pro Plus software version 6 (Media Cybergenetics, United States) (Sen et al., 2012; Al-Fataftah and Abdelqader, 2014).

### 16S rDNA Gene Sequencing and Analysis

Cecal contents were collected for microbial analyses after 21 days of feeding. Total genomic DNA from samples was extracted using Hipure Soil DNA Kit (Magen, Guangzhou, China) according to the manufacturer’s protocols. DNA concentration was monitored by Qubit 3.0 Fluorometer. The V3 and V4 hypervariable regions of prokaryotic 16S rDNA were amplified using forward primer CCTACGGRRBGCASCAGKVRVGAAT and reverse primer GGACTACNVGGGTWTCTAATCC. DNA libraries (10 nM) were multiplexed and loaded onto an Illumina MiSeq or NovaSeq instrument according to manufacturer’s instructions (Illumina, San Diego, CA, United States). After quality filtering, the chimeric sequence was removed, and the final sequence was used for operational taxonomic unit (OTU) clustering using Vsearch clustering (1.9.6) with 97% sequence similarity. The Ribosomal Database Program (RDP) classifier Bayesian algorithm of the OTU species taxonomy was then used to analyze representative sequences and under different species classification levels, statistical community compositions of each sample were performed. Based on the OTU analysis results and by using the method of random flattening sample sequences, the alpha diversity indices of ace, shannon, chao 1, and simpson were calculated by Qiime (1.9.1) software to reflect the species richness and diversity.

### Susceptibility of Chickens to *E. coli*

All the chickens were intraperitoneal injections with 0.5 mL of bacterial suspension (10^7^ cfu/mL). The unchallenged control received 0.5 mL saline. After infection for 1 and 3 days, *E. coli* counts were determined by analyzing the hearts, livers, spleens, lungs, and kidneys from five chickens per group. The collected tissue samples were mixed with PBS (1 mL/g) and were ground into tissues homogenate. Then tissue homogenates were 10-fold diluted with PBS, and plated onto Luria-Bertani agar to calculate the *E. coli* CFU. The survival rate of chickens was calculated at the end of the experiment.

### Effect of Dietary *B. subtilis* on Transcript Level of Immunity-Related Genes

To measure the expression of immunity-related genes, RNA was extracted from spleen samples using TRIzon Reagent (CoWin Biosciences, Beijing, China). Reverse transcription was performed with HiScript^*R*^ II Q Select RT SuperMix for real-time polymerase chain reaction (qPCR + gDNA wiper) (Vazyme, Nanjing, China). β-actin and immune-related gene primers for quantitative qPCR oligonucleotides are listed in Table 2. qPCR was performed with the Applied Biosystems 7500 Fast Real-Time PCR System (Applied Biosystems, CA, United States). qPCR reactions were performed in a total volume of 20 μL with the SYBR Green PCR kit (Transgen Biotech Co., Ltd., Beijing, China). qPCR reactions were run using a specific cycling protocol: 30 s at 94°C; 40 cycles of 5 s at 94°C; 34 s at 60°C; and finally with a dissociation curve. The relative expressions of mRNA were calculated based on the 2^–ΔΔCt^ method. Target gene mRNAs were normalized to β-actin of each sample. Each sample was run in triplicate. The results were expressed as the *B. subtilis* vs. the control group.

**TABLE 2:** Primers used in this study.

Primer name	Sequence (5′-3′)	GenBanknumber
*Tlr4* F	AGTCTGAAATTGCTGAGCTCAAAT	AY064697
*Tlr4* R	GCGACGTTAAGCCATGGAAG	
*Mhc II-*α F	TGGGATCCTCCGTCCTGAAGCCGCAC	EF554707.1
*Mhc II-*α R	GCGTCGACTCAGAGCAGCCCCGGTT	
*Myd88* F	TGATGCCTTCATCTGCTACTG	EF011109
*Myd88* R	TCCCTCCGACACCTTCTTTCTA	
*NF-*κ*B* F	CAGCCCATCTATGACAACCG	NM-205129
*NF-*κ*B* R	TCCCTGCGTCTCCTCTGTGA	
*Ifn-*α F	ATGCCACCTTCTCTCACGAC	EU367971
*Ifn-*α R	AGGCGCTGTAATCGTTGTCT	
*Il1*β F	GTGAGGCTCAACATTGCGCTGTA	NM204524.1
*Il1*β R	TGTCCAGGCGGTAGAAGATGAAG	
*Il6* F	TCTGTTCGCCTTTCAGACCTA	AJ309540
*Il6* R	GACCACCTCATCGGGATTTAT	
*Il8* F	ATGAACGGCAAGCTTGGAGCTG	DQ393272.2
*Il8* R	TCCAAGCACACCTCTCTTCCATCC	
β*-actin* F	GAGAAATTGTGCGTGACATCA	L08165.1
β*-actin* R	CCTGAACCTCTCATTGCCA	

### Statistical Analysis

The non-parametric Mann–Whitney *U* test was conducted to examine significant differences between *B. subtilis* and control group using the SPSS computer software (SPSS Inc., Chicago, IL, United States). The survival rate of chickens was calculated by Kaplan-Meier method and the significant difference was analyzed by Log-rank test. A value of *P* < 0.05 was considered significant.

## Results

### Growth Performance and Serum Immunoglobulin

**FIGURE 1:**
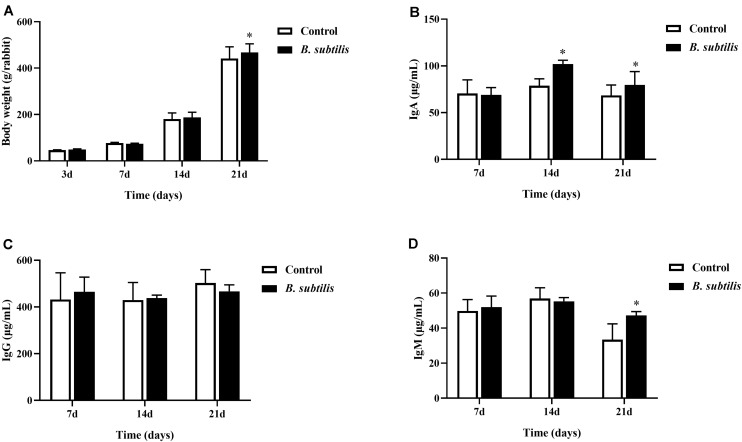
Effects of dietary *Bacillus subtilis* on growth performance and serum immunoglobulin of chickens. **(A)** The body weights were measured at 3, 7, 14, and 21 days after feeding *B. subtilis*. The concentrations of IgA **(B)**, IgG **(C)**, and IgM **(D)** were measured at 7, 14, and 21 days after feeding *B. subtilis*. Bars were expressed as means ± standard deviations (*n* = 5). *P < 0.05.

The chickens fed diet with *B. subtilis* had higher body weight than chickens fed standard control diet, especially at 21 days of feeding (Figure 1A). After 14 and 21 days of feeding, serum IgA of chickens fed with *B. subtilis* were significantly higher (*P* < 0.05) than that of controls (Figure 1B). However, no significant differences was observed in serum IgG between chickens fed with *B. subtilis* diets and the controls (Figure 1C). Chickens fed diets containing *B. subtilis* showed higher IgM levels than the controls at 21 days (Figure 1D).

### Small Intestinal Morphology

To gain better insight into the effect of *B. subtilis* on intestinal morphology, the duodenum, jejunum, and ileum were collected for histomorphological analyses (Supplementary Figure 1). As shown in Table 3, chickens fed with *B. subtilis* had greater villus heights (VH) of jejunum (*P* < 0.05) and ileum at 21 days. The crypt depth (CD) of the duodenum, jejunum (*P* < 0.05), and ileum were shallower in chickens fed with *B. subtilis* than those fed with the control diet. The villus height: crypt depth (VH/CD) ratio of the duodenum, jejunum, and ileum were higher (*P* < 0.05) in chickens fed with *B. subtilis* than the control.

**TABLE 3:** Effects of dietary *Bacillus subtilis* on intestinal villus-crypt system.

		Control	*Bacillussubtilis*
**Duodenum**			
	Villus height (μm)	1507.67 ± 202.30	1579.00 ± 54.69
	Crypt depth (μm)	194.00 ± 8.54	187.67 ± 12.93
	VH/CD	7.77 ± 1.25^a^	8.41 ± 0.51^b^
**Jejunum**			
	Villus height (μm)	749.33 ± 106.44^a^	903.00 ± 107.87^b^
	Crypt depth (μm)	210.08 ± 33.99^a^	141.33 ± 17.80^b^
	VH/CD	3.57 ± 0.61^a^	6.39 ± 0.77^b^
**Ileum**			
	Villus height (μm)	606.17 ± 71.77	555.75 ± 15.09
	Crypt depth (μm)	154.75 ± 28.03	119.56 ± 15.87
	VH/CD	3.92 ± 0.56^a^	4.65 ± 0.33^b^

*Data were expressed as means ± standard deviations (n = 5). ^a,b^Values with different superscripts in the same row differ significantly (P < 0.05).*

### Taxonomic Composition of Intestinal Microbiota

**FIGURE 2:**
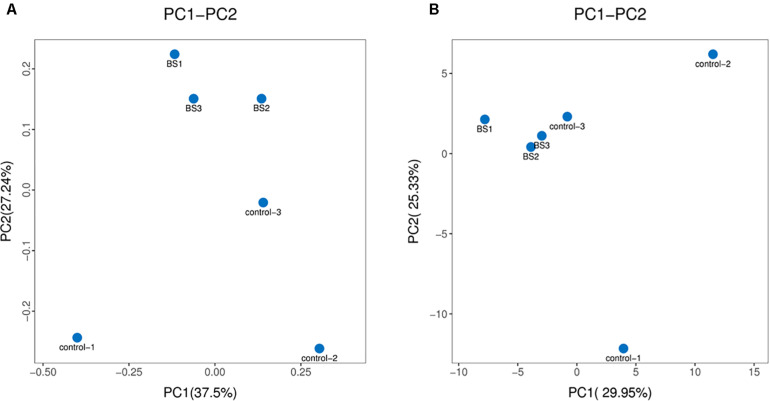
β-diversity analysis of microbial communities. **(A)** Principal co-ordinates analysis (PCoA) plot based on Brary-Curtis distances (*n* = 3). **(B)** Principal component analysis (PCA) plot based on the distribution of bacterial community.

To determine how *B. subtilis* regulates intestinal microbiota, 16S rDNA sequences of cecal contents were analyzed at 21 days. The 16S rDNA sequences have been deposited in GenBank (SRA accession: PRJNA612427). A total of 1,627,645 reads were obtained from all samples with 53.13% (G + C). Diets containing *B. subtilis* showed no effects in α-diversity of ace, chao 1, shannon, and simpson indices (Table 4). As shown in Figure 2, PCoA and PCA plots showed a good separation of cecal microbiota communities between two groups.

**TABLE 4:** α-diversity of microbial community structure.

Sample	Ace	Chao1	Shannon	Simpson
*Bacillus subtilis*	143.06 ± 9.23	144.1411.14	5.26 ± 0.32	0.94 ± 0.02
Control	146.37 ± 1.80	149.838.17	5.21 ± 0.25	0.95 ± 0.00

*Data were expressed as means ± standard deviations (n = 3).*

**FIGURE 3:**
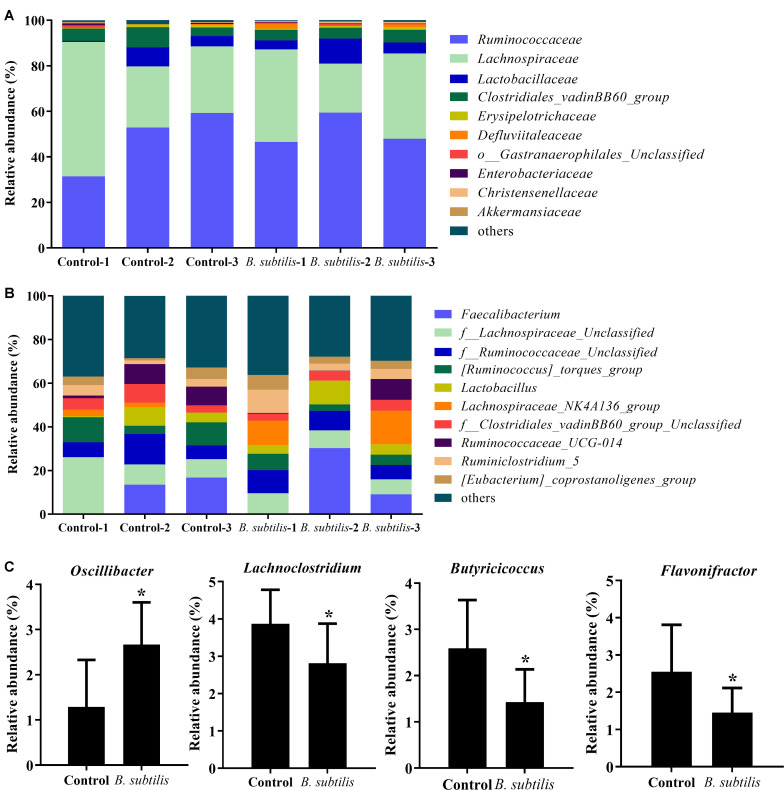
Effects of dietary *Bacillus subtilis* on cecal microbes at different taxonomic levels in chickens. **(A)** Relative abundance of the top 10 bacteria at the family level. **(B)** Relative abundance of the top 10 bacteria at the genus level. **(C)** Relative abundance of genera *Oscillibacter*, *Lactobacillus, Butyricicoccus*, and *Faecalibacterium*. Bars were expressed as means ± standard (*n* = 3). *P < 0.05.

At family level, the main families were *Ruminococcaceae*, *Lachnospiraceae*, *Lactobacillaceae*, *Clostridiales_vadinBB60_gro up*, *Erysipelotrichaceae*, *Defluviitaleaceae*, *o__Gastranaerophil ales_Unclassified*, *Enterobacteriaceae, Christensenellaceae*, and *Akkermansiaceae* in chickens fed with *B. subtilis*. The relative abundance of *Ruminococcaceae* was increased after feeding with the *B. subtilis* diet (Figure 3A). At the genus level, the dominant species *Faecalibacterium* (13.18%) and *Lactobacillus* (6.62%) in chickens fed with the *B. subtilis* diet were significantly higher than that of controls (Figure 3B). Moreover, the relative abundance of *Oscillibacter* was increased after feeding *B. subtilis*, while the percentage of *Lachnoclostridium*, *Butyricicoccus*, and *Flavonifractor* showed a significant decrease (Figure 3C; *P* < 0.05).

### Effect of Dietary *B. subtilis* on Cytokine Transcript Levels of the Spleen

**FIGURE 4:**
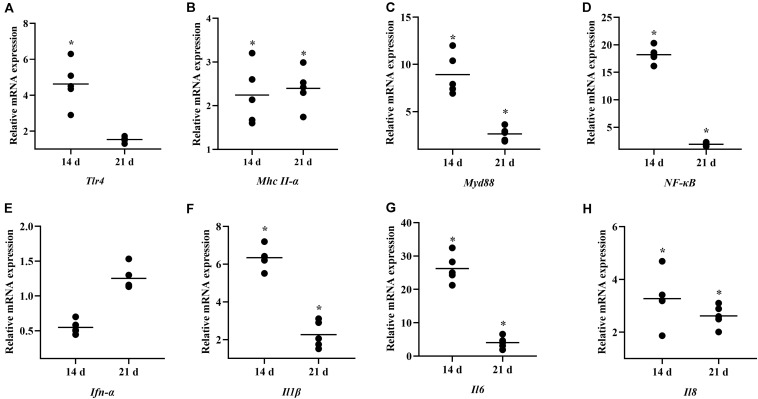
Effects of dietary *Bacillus subtilis* on the levels of transcripts of immune-related genes. **(A)** *Tlr4*, **(B)** *Mhc II-*α, **(C)** *Myd88*, **(D)** *NF-*κ*B*, **(E)** *Ifn-*α, **(F)** *Il1*β, **(G)** *Il6*, **(H)** *Il8*. The relative mRNA expression represent the target gene expressions of diets with *B. subtilis* vs. controls. Bars were expressed as geometric mean (*n* = 5). *P < 0.05.

To investigate the role of *B. subtilis* in innate immunity, the transcript levels of immunity-related genes in the spleen were conducted by qPCR at 14 and 21 days. Our results showed that the major pro-inflammatory factors in the chickens were upregulated after feeding probiotics. Expressions of toll-like receptor 4 (*Tlr4*) and major histocompatibility complex (*Mhc*) *II-*α were significantly upregulated by 4.35- and 2.13-fold, respectively, at 14 days (Figures 4A,B). The signal transducing adaptor protein *Myd88* was significantly upregulated by 8.90- and 2.77-fold at 14 and 21 days, respectively (Figure 4C). Next, the expression of the downstream signaling molecule, nuclear factor kappa B (*NF-*κ*B*) was also upregulated after feeding the diet with *B. subtilis* (Figure 4D). However, interferon (*Ifn*)*-*α expression showed no significant difference in chickens fed the *B. subtilis* diet compared to the controls (Figure 4E). At 14 and 21 days, the proinflammatory cytokines *Il1*β, *Il6*, and *Il8* showed obvious changes in chickens fed the *B. subtilis* (Figures 4F–H).

### Survival Rate and *E. coli* Content

**FIGURE 5:**
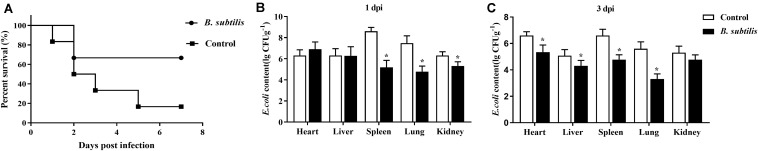
Effects of dietary *Bacillus subtilis* on disease resistance against *Escherichia coli*. **(A)** The survival rate of chickens after infection with *E. coli* (*n* = 10). **(B,C)** *E. coli* contents of infected chickens at 1 and 3 dpi, respectively (log_10_ CFU g^– 1^). Bars were expressed as means ± standard (*n* = 5). *P < 0.05.

As shown in Figure 5A, the survival rate of chickens fed with the *B. subtilis* diet was 66.67%, which was significantly higher than in the controls. The chickens in the control group died at 10 h after challenge. The mortality rate of control group reached the highest at 2 days post-infection (dpi) and then decreased. At 5 dpi, the survival rate of chickens fed with *B. subtilis* was 16.67%, and none of the chickens died after 6 dpi. However, the mortality rate of the probiotic group was 33.33%, and none of the chickens died after 3 dpi. The chickens resumed the feed intake and drinking water at 3 dpi. Taken together, in the *B. subtilis* group, the time of death was delayed, and the number of deaths were fewer after *E. coli* infection.

*E. coli* contents in heart, liver, spleen, lung, and kidney were detected at 1 and 3 dpi. As shown in Figures 5B,C, the amount of *E. coli* in the test tissues of chickens fed with *B. subtilis* were lower than that of controls. Especially, *E. coli* contents in spleens and lungs of chickens fed with probiotics were 1.5 × 10^5^ cfu/g and 6.1 × 10^4^ cfu/g, which were lower than 1.7 × 10^8^ cfu/g and 3.3 × 10^7^ cfu/g of controls, respectively (*P* < 0.05). At 3 dpi, the number of *E. coli* in tested tissues had declined compared with 1 dpi (Figure 5C).

### Expression of Innate Immune-Related Genes in the Spleen of the Infected Chickens

**FIGURE 6:**
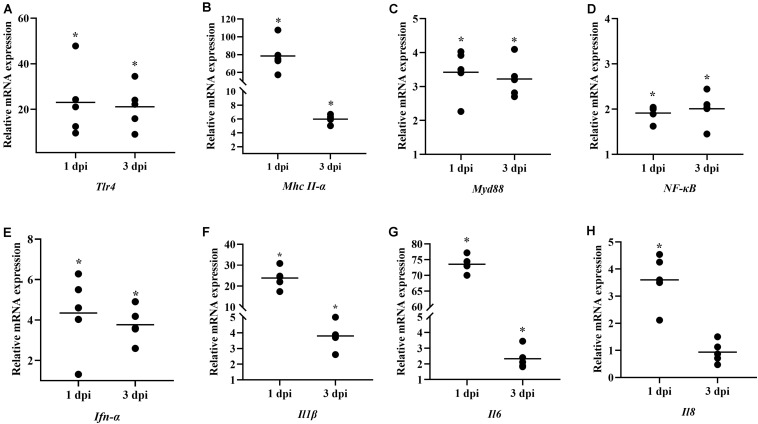
Effects of dietary *Bacillus subtilis* on transcript levels of immune-related genes after infection with *E. coli*. **(A)** *Tlr4*, **(B)** *Mhc II-*α, **(C)** *Myd88*, **(D)** *NF-*κ*B*, **(E)** *Ifn-*α, **(F)** *Il1*β, **(G)** *Il6*, **(H)** *Il8*. The relative mRNA expression represents the target gene expression of the diet containing *B. subtilis* rather than that of the controls after infection with *E. coli*. Bars were expressed as geometric mean (*n* = 5). *P < 0.05.

We next studied the expression of innate immune-related genes induced by *B. subtilis* after *E. coli* infection. The induction of innate immunity-related genes were expressed more frequently in spleens at 1 dpi, and the up-regulation decreased at 3 dpi. Administration of *B. subtilis* significantly increased the *Tlr4*, *Myd88*, and *NF-*κ*B* by 2–22-fold (Figures 6A,B,D, respectively). The expression of *Mhc II-*α was upregulated 79.31-fold on 1 dpi (Figure 6C). The pro-inflammatory cytokines, *Ifn-*α, *Il1*β, *Il6*, and *Il8* mRNA levels showed elevated expressions compared with control chickens (Figures 6E–H). In particular, the expression of *Il1*β and *Il6* were upregulated by 24.33- and 74.40-fold at 1 dpi, respectively. At 3 dpi, the expressions of *Il1*β and *Il6* were up-regulated by 3.80- and 2.31-fold, respectively (Figures 6F,G).

## Discussion

Increasing evidence supports the concept that dietary *B. subtilis* can promote the growth performance of animals (Lee S.H. et al., 2014; Musa et al., 2019; Sokale et al., 2019). However, some other studies showed that there is no relationship of *B. subtilis* with average daily gain and feed conversion ratio (Lee K.W. et al., 2014; Zhang et al., 2017). It has been demonstrated that probiotic functions are affected by environmental temperature, probiotic strains, administration levels, and animal species. In this study, we investigated growth performance, serum immunoglobulins, intestinal homeostasis, immune responses, and disease resistance of chickens with *B. subtilis* strains BG5 and BYS2 addition.

In this study, administration of *B. subtilis* into diets caused an increase in chicken body weight gain. Higher digestive and absorption of nutrients in animals fed with probiotics might be due to improved intestinal morphology, modulated gut environment, and stimulated immune responses (Choi et al., 2011). The changes of intestinal morphology including the increase in VH and VH/CD ratio indicated an improved absorptive surface area and digestive and absorption capacities (Sen et al., 2012). Crypts are the sites of proliferation and differentiation of intestinal epithelial cells that promote villus growth. Similarly with the previous study the previous study (Ma et al., 2018), our results showed that chickens fed with *B. subtilis* had greater VH, shallower CD, and higher VH/CD ratio in the jejunum, which might also explain the elevated growth performance in chickens. Furthermore, growth performance improvement is related to nutrients and extracellular digestive enzymes produced by *B. subtilis* (Sahu et al., 2008). In our previous research, we have proved that *B. subtilis* used in this study had good performance in protease activities.

Serum IgA and IgM increases found in chickens fed with *B. subtilis* diets in the present study reflected an improvement in immune function. These results are in agreement with previous research in which administration of *B. subtilis* promoted the increase in IgA concentrations in pigs (Lee S.H. et al., 2014). The gastrointestinal tract is particularly sensitive to stress and especially can cause changes in normal intestinal microbiota. It is widely believed that the main reason probiotic-related growth improvement performance and intestinal morphology is intestinal microbiota modulation (Crisol-Martínez et al., 2017). In the present study, administration of *B. subtilis* modified the relative abundance of cecal microbiota. In particular, the relative abundance of *Ruminococcaceae*, *Faecalibacterium*, *Lactobacillus*, and *Oscillibacter* were increased after feeding a *B. subtilis* diet. The *Ruminococcaceae* species are positively related to body weight, and they play a vital role in fiber degradation and butyrate production (Zhao et al., 2017; Fang et al., 2019). *F. prausnitzii* is a functionally important commensal bacterium, which has an impact on the physiology and health of host. The amount of *F. prausnitzii* is negatively associated with inflammatory bowel disease and colorectal cancer. *F. prausnitzii* is the only species identified in the genus *Faecalibacterium* (Benevides et al., 2017; Ferreira-Halder et al., 2017). As probiotics, *Lactobacillus* and *B. subtilis* have been suggested as the greatest potential therapy. *Oscillibacter* is a newly discovered genus that plays a crucial role in maintaining mucosal homeostasis and anti-inflammatory functions (Lopetuso et al., 2013; Wang et al., 2018). In contrast, *Flavonifractor* was reported to induce oxidative stress and inflammation (Coello et al., 2019). *Lachnoclostridium* and *Butyricicoccus* are related to chronic low-grade inflammation and metabolic diseases and negatively related to body weight in human and animals (Guo et al., 2018; Fang et al., 2019; Kong et al., 2019). Hence, the relevant increase in *Ruminococcaceae* and *Lactobacillus* and decrease in *Lachnoclostridium* and *Butyricicoccus* could partially explain the increase in body weights in chickens fed with *B. subtilis*. Taken together, our results indicate that diets with *B. subtilis* can improve growth performance, modulate intestinal microbiota, and maintain intestinal homeostasis.

It is generally believed that probiotics exert opposite effects on pathogenic bacteria, including inhibition rather than stimulation of proinflammatory responses (Jijon et al., 2004). However, several studies emphasized the importance of relating “physiological inflammation” that is caused by symbiotic microbiota immune system development and the response to intestinal pathogens. Accordingly, the pro-inflammatory response induced by probiotics is currently under exploration. TLRs, as a type of pattern recognition receptor, can activate immune responses and regulate inflammatory responses. In the previous study, oral administration of probiotics caused an increase in *Tlr3* and *Tlr4* gene expressions (Cario and Podolsky, 2000). Moreover, changes in the intestinal microbiota due to the *B. subtilis* addition may have also led to the different activation of pattern recognition receptors. Our results showed that after dietary administration of *B. subtilis* increased the mRNA expression level of *Tlr4*. *Myd88* is a kind of adapter protein that plays an effective role in activating the downstream *NF-*κ*B* signaling pathway. In the present study, application of *B. subtilis* significantly increased the expression of *Myd88* and *NF-*κ*B*. Similarly, diets with *Saccharomyces boulardii* and *B. subtilis* activated the innate immune response that was dependent on the *Myd88* signaling pathway (Rajput et al., 2017). *NF-*κ*B* plays a beneficial role in protecting the development of systemic inflammation (Chen et al., 2003). In this study, dietary with *B. subtilis* significantly increased the mRNA expression levels of proinflammatory cytokines (*Il1*β, *Il6*, and *Il8*). Our results were similar as those previous study in which the probiotic VSL#3 activated *Tlr9*-mediated activation of *NF-*κ*B* and promoted proinflammatory cytokines *Il6* and *Il12* (Rachmilewitz et al., 2004). Taken together, diets containing *B. subtilis* could trigger *Tlr4* and activate proinflammatory cytokine production to develop innate immunity responses in chickens.

Probiotics can help animals resist pathogenic bacteria infections. In a previous study, *B. subtilis* DSM 32315 significantly enhanced the disease resistance after a necrotic enteritis challenge (Whelan et al., 2019). In the current study, the survival rate of chickens fed with *B. subtilis* was higher than that of controls after infected with *E. coli.* The *E. coli* contents in the hearts, livers, spleens, lungs, and kidneys of chickens fed with *B. subtilis* were lower than that of controls. This conclusion was supported by growth performance promotion, increases in serum immunoglobulins, promotion of intestinal homeostasis, and enhances immune responses. It is worth noting that the major pro-inflammatory factors (*Ifn-*α, *Il1*β, *Il6*, and *Il8*) were upregulated in the *B. subtilis* feeding group after being challenged with *E. coli*. *Il1*β is the major proinflammatory cytokine, which induces the production of itself, other proinflammatory cytokines and chemokines (such as *Il6*, *Il8*, and *Tnf-a*). In return, they can activate inflammation and recruit neutrophils and macrophages which can phagocytize and kill bacteria. Previous study showed that probiotics administration almost complete prevent mucosal damage, with preservation of the normal villi morphology and minimal inflammatory infiltration. The results were related to activation of NF-κB and production of TNF-α, coupled with restitution of normal intestinal epithelial barrier function (Pagnini et al., 2010). In fact, activation of NF-κB plays a beneficial role in epithelial cells. Activation of NF-κB has a similar protective effect on the development of systemic inflammation, but not for local injury (Chen et al., 2003). The proinflammatory cytokine, is fundamental in establishing the inflammatory disease can have an opposing, protective effect during disease initiation. Because proinflammatory cytokines (such as IFN-γ, IL-6, and TNF-α) are part of the innate immune system, one hypothesis is that proinflammatory cytokines are partly directed against luminal antigens (bacterial or food antigens) that would otherwise threaten mucosal integrity. We deduced that *B. subtilis* prevent bacterial disease by mechanisms involving activation of *NF-*κ*B*, and maintain the integrity of the intestinal epithelium.

## Conclusion

In conclusion, diets containing *B. subtilis* appear to improve growth performance, serum immunoglobulin levels, intestinal homeostasis, immune responses, and disease resistance in chickens. In addition, *B. subtilis* can trigger *Tlr4* and activate proinflammatory responses in chickens. Our results indicated that *B. subtilis* may be an antibiotic alternative approach for controlling bacterial infection.

## Data Availability Statement

The original contributions presented in the study are publicly available. This data can be found here: https://www.ncbi.nlm.nih.gov/sra/?term=PRJNA612427.

## Ethics Statement

The animal study was reviewed and approved by the Committee on the Ethics of Animal Experiments of Yangzhou University.

## Author Contributions

MG and ML designed and conducted the study, performed most of the experiments, and wrote the manuscript. CZ performed the calculation with support from XZ. YW discussed the results and revised the manuscript. All authors contributed to the article and approved the submitted version.

## Conflict of Interest

The authors declare that the research was conducted in the absence of any commercial or financial relationships that could be construed as a potential conflict of interest.

## Supplementary Material

FIGURE S1 Effects of dietary *Bacillus subtilis* on intestinal morphology of chickens. The representative photomicrographs of hematoxylin and eosin (H&E)-stained sections of intestinal sections: **(A)** Duodenum, **(B)** Jejunum, and **(C)** Ileum in chickens fed with *B. subtilis.* **(D–F)** Show the duodenum, jejunum, and ileum of chickens fed with the control diet, respectively, at 40× original magnification.
